# Refinement of Imaging Predictors of Recurrent Events following Transient Ischemic Attack and Minor Stroke

**DOI:** 10.1371/journal.pone.0065752

**Published:** 2013-06-21

**Authors:** Myles Horton, Jayesh Modi, Shiel K. Patel, Andrew M. Demchuk, Mayank Goyal, Michael D. Hill, Shelagh B. Coutts

**Affiliations:** 1 Department of Clinical Neurosciences, University of Calgary, Calgary, Alberta, Canada; 2 Department of Radiology, University of Calgary, Calgary, Alberta, Canada; 3 Department of Community Health Sciences, University of Calgary, Calgary, Alberta, Canada; 4 Department of Medicine, University of Calgary, Calgary, Alberta, Canada; 5 Hotchkiss Brain Institute, University of Calgary, Calgary, Alberta, Canada; University of Cambridge, United Kingdom

## Abstract

**Background:**

TIA and minor stroke have a high risk of recurrent stroke. Abnormalities on CT/CTA and MRI predict recurrent events in TIA and minor stroke. However there are many other imaging abnormalities that could potentially predict outcome that have not been assessed in this population. Also the definition of recurrent events used includes deterioration due to stroke progression or recurrent stroke and whether imaging is either of these is not known.

**Aims:**

To improve upon the clinical, CT/CTA and MRI parameters that predict recurrent events after TIA and minor stroke by assessing further imaging parameters. Secondary aim was to explore predictors of stroke progression versus recurrent stroke.

**Methods:**

510 consecutive TIA and minor stroke patients had CT/CTA and most had MRI. Primary outcome was recurrent events (stroke progression or recurrent stroke) within 90 days. Further imaging parameters were assessed for prediction of recurrent events (combined outcome of stroke progression and recurrent stroke). We also explored predictors of symptom progression versus recurrence individually.

**Results:**

36 recurrent events (36/510, 7.1% (95% CI: 5.0–9.6)) including 19 progression and 17 recurrent strokes. On CT/CTA: white matter disease, prior stroke, aortic arch focal plaque≥4 mm, or intraluminal thrombus did not predict recurrent events (progression or recurrent stroke). On MRI: white matter disease, prior stroke, and microbleeds did not predict recurrent events. Parameters predicting the individual outcome of symptom progression included: ongoing symptoms at initial assessment, symptom fluctuation, intracranial occlusion, intracranial occlusion or stenosis, and the CT/CTA metric. No parameter was strongly predictive of a distinct recurrent stroke.

**Conclusions:**

There was no imaging parameter that could improve upon our original CT/CTA or MRI metrics to predict the combined outcome of stroke progression or a recurrent stroke after TIA and minor stroke. We are better at using imaging to predict stroke progression rather than recurrent stroke.

## Introduction

Transient ischemic attacks (TIAs) and minor strokes carry a high-short term risk of recurrent stroke. Much previous work has been completed to determine the clinical and imaging factors that increase the risk of developing a disabling stroke following a TIA or minor stroke. There are several clinical and event related factors that increase the risk of stroke after TIA [1]. Imaging also plays a role in predicting outcomes in these patients [2]–[4].

We recently showed in the CATCH study that predefined radiographic abnormalities on CT/CTA and MRI predicted recurrent events after TIA and minor stroke [5]. Specifically, the study assessed the predictive value of CT/CTA abnormalities that were defined *apriori*: acute ischemia on CT, intracranial or extracranial occlusion or stenosis ≥50% (the CT/CTA positive metric), and MRI: acute lesion on diffusion weighted imaging (DWI). In the multivariable analysis, only the CT/CTA abnormalities predicted recurrent events. This main CATCH publication presented only the analysis of the simple predefined abnormalities on CT/CTA and MRI (DWI) and the literature would suggest that, particularly in the longer term, there are other imaging parameters that may add to the prediction of recurrent events. This includes white matter disease [6]–[8], previous strokes on imaging [6], microbleeds [9]–[10], and aortic arch atheroma [11]. The current study sought to see if the inclusion of these additional imaging parameters from the CT/CTA or MRI can better predict recurrent events after TIA and minor stroke. Further a recent publication found that symptom fluctuation predicts recurrent events and we were interested to see if this was true in our population [12].

Additionally the definition of recurrent events in the CATCH study included the outcomes of symptom progression (worsening due to the reason that the patient presented in the first place; e.g. collateral failure in the setting of an intracranial occlusion in the first 24 hours of admission or capsular warning syndrome etc.) and a distinct recurrent stroke (e.g. clinical symptoms from a second embolus in a different arterial territory). These were included together in the CATCH study. Most publications have not differentiated these outcomes as they did not have the necessary imaging information and both of these outcomes are clinically relevant and impact outcome. Stroke progression and recurrence likely have different pathophysiologies and we sought to complete an exploratory analysis on clinical and imaging predictors of symptom progression versus recurrent stroke to see if we can predict one better than the other.

We hypothesized that we could improve the clinical, CT/CTA and MRI parameters that predict recurrent events after TIA and minor stroke. Secondarily, we sought to complete an exploratory analysis of predictors of symptom recurrence versus progression.

## Methods

Consecutive patients aged at least 18 years presenting with a high risk TIA focal weakness or speech disturbance lasting at least five minutes or minor ischemic stroke (NIHSS score of 3 or less) who were referred to the stroke team at Foothills Medical Centre, were prospectively considered for enrolment. Patients were examined by a stroke neurologist and had a CT brain and CTA of the circle of Willis and neck within 24 hours of symptoms onset. The majority of patients also had a stroke MRI performed. Exclusion criteria included acute treatment with a thrombolytic drug, pre-morbid mRS of 2 or greater, and the presence of a serious co-morbid illness that would likely result in death within three months. The local ethics committee – The Calgary Health Research Ethics Board - associated with the Foothills Medical Centre and the University of Calgary approved this protocol and each patient provided written informed consent. Detailed baseline clinical and outcome data was collected for each participant. Above standard clinical data we sought to review the role of fluctuation in the prediction of recurrent events. Fluctuation was prospectively defined as having occurred if a patient deteriorated then improved on more than one occasion with symptoms referable to the same brain location. This was rated at the time of the initial physician neurological assessment and was based either on the clinical history or on clinical examination (if the patient was still fluctuating at the time of initial assessment). Fluctuation later than the time of initial assessment was not recorded. Any event that occurred more than 24 hours before the initial assessment was not considered in the assessment of fluctuation. Patients received routine clinical care including antihypertensive and lipid-lowering therapy at the discretion of the treating physician. Ongoing symptoms were assessed at the time of first assessment by the stroke neurology team in the emergency department. Ongoing symptoms were defined as the patient having any deficit measureable on the standard bedside neurological examination. All individuals were trained in the neurological examination and were completed by either a stroke neurologist or stroke fellow.

### Imaging

Detailed imaging information has been previously described [5]. CT imaging was performed on a Siemens 64 slice scanner. Standard whole brain axial CT was performed with a sequential (non-helical) technique at 5-mm slice thickness. CT was immediately followed by CTA from aortic arch to skull vertex with a helical scan technique at 0.6 mm thickness. MRIs were completed on either a GE 3T scanner or a Siemens 1.5T MR scanner. All imaging was assessed by a neuroradiologist who remained blinded to the results of the other imaging modality and was given information regarding the clinical symptoms only. The CT/CTA metric previously tested was: acute ischemia on CT or CTA showing symptomatic intracranial or extracranial occlusion or stenosis ≥50% (any narrowing >50% was included as part of the CTA metric, whether it was felt to be atherosclerotic or a partial occlusion). MRI was assessed for acute or hyperacute lesions on DWI (DWI positive) using axial DWI, ADC and FLAIR sequences.

In this study we *apriori* chose to assess the following additional imaging parameters: a) CT: remote infarcts (including both cortically based or cavitating lacunar infarcts), white matter chronic ischemia, intraluminal thrombus [13], focal aortic arch plaque ≥4 mm on CTA; b) MRI: remote infarcts on FLAIR (including both cortically based or cavitating lacunar infarcts), microbleeds on gradient echo (GRE), and any age-related white matter disease on FLAIR (any area of hyperintensity in the white matter on FLAIR not related to a current or previous stroke). Microbleeds were defined on GRE as hypointense rounded lesions <5 mm in the brain parenchyma [14].

A recurrent event was defined as a functional deterioration in neurological status of vascular origin lasting 24 hours or longer or a new sudden focal neurological deficit of vascular origin lasting at least 24 hours (that was not felt to be secondary to other non vascular factors: drugs, fever, infection) [1], [15]–[16]. Deterioration was assessed by a clear worsening in the deficits as compared to the baseline assessment, but did not necessarily require a change in the NIHSS – eg worsening hand weakness not captured by the NIHSS. Repeat imaging was mandated for all recurrent events (CT at minimum and MRI recommended). All recurrent events were reviewed in detail by a panel of three physicians that included two stroke neurologists (SBC and AMD) and a neuroradiologist (MG). Recurrent events captured using this standard definition includes both progression of the presenting event (symptom progression) and a distinct second recurrent event (recurrent stroke). Recurrent events were categorised as progression or recurrent stroke using clinical and imaging information. For example, a patient who worsened as a result of a deterioration related to the presenting event would be rated as progression and those with a second embolus would be rated as a recurrent stroke [17]. For a patient to be labelled as progression the clinical worsening had to be clinically referable to the same territory as the baseline symptoms with no imaging evidence of an infarct in a location separate from the baseline imaging. If it were not clear regarding the mechanism of deterioration then the event would be rated as a recurrent stroke. Also to be rated as a progression the deterioration had to be temporally related to the presenting event. There was no absolute cut off in time that would definitively make an event recurrence versus progression, however it was expected that progression would be unlikely after 72 hours from onset [18]. Patients were followed for 90 days from symptom onset.

### Statistical Analysis

Statistical analysis was completed with Stata (version 12). Imaging parameters other than those included in the original analysis [5] were assessed for prediction of overall recurrent events to attempt to refine the CT/CTA metric and DWI positive MRI. 420 patients had an MRI as well as CTA. For the other patients the missing DWI values were imputed as previously described [5]. The risk of recurrent events was assessed using standard clinical variables, symptom fluctuation, and the imaging parameters detailed above. Univariable analysis was conducted using a simple Cox proportional hazards model. A multivariable Cox proportional hazard model was developed using predefined variables that were chosen either from statistically significant results (p<0.05) from the univariable analysis or that have been previously shown to predict recurrent stroke as part of the ABCD^2^ score [1]. Variables were removed in a stepwise fashion if not predictive of recurrent stroke. The proportional hazards assumption was tested and found to be valid. An exploratory univariable analysis was subsequently completed using a simple Cox proportional hazards model for predictors of symptom progression versus recurrent stroke.

## Results

Five hundred ten patients were enrolled into the study during a period of 29 months. Follow up information was available in 98% (499/510). Baseline demographics are shown in Table 1. Four hundred twenty (82%) patients received an MRI for their initial event and 243 of these were DWI-positive (58%; 95% CI, 53–63). There were 36 recurrent events (7.1%; 95% CI, 5.0–9.6). Of these, 19 events were progression and 17 were recurrent strokes. Median time to recurrent event was 1 day (IQR 7.5).

**Table 1:** Baseline demographics.

	N = 510
Age (years) [median, (IQR)]	69, (21.2)
Gender – male [n, %]	302 (59)
Baseline NIHSS [median, IQR]	1 (1)
Diabetes mellitus [n, %]	77 (15)
Hypertension [n, %]	285 (56)
Current smoker [n, %]	79 (15)
Symptom to baseline CT (hours) [median, IQR]	4.9 (2.1)
Symptom to baseline MRI (hours) [median, IQR]	17.6 (12)
Treated with aspirin [n,%]	435 (86)
Treated with aspirin and clopidogrel [n,%]	150 (30)
Treated with statin [n,%]	303 (60)

Table 2 shows the analysis attempting to refine the clinical and imaging predictors of recurrent events (including events subcategorized as symptom progression or recurrent stroke) The clinical variable of symptom fluctuation was predictive of recurrent events (HR 2.3, 95% CI: 1.05–5.0). None of the additional imaging variables were predictors of recurrent events. Symptom fluctuation remained significant in the multivariable cox proportional hazards model: CT/CTA at risk metric (HR 4.2, 95% CI 2.1–8.5, p<0.001), Symptom fluctuation (HR 2.7, 1.2–5.9 p = 0.015).

**Table 2:** Effect of new imaging parameters on combined recurrent events (including symptom progression and a distinct recurrent stroke).

Variable	Combined RecurrentEvent, % (no./No.)	No Combined RecurrentEvent, % (no./No.)	Hazard Ratio (95% CI)
**CT/CTA Imaging**			
White matter disease	25 (9/36)	29 (136/463)	0.8 (0.4–1.8)
Prior imaging stroke	31 (11/36)	21 (95/463)	1.7 (0.8–3.4)
Aortic arch focal plaque ≥4 mm	9 (3/35)	5 (24/459)	1.6 (0.5–5.2)
ICA intraluminal thrombus	3 (1/36)	2 (11/463)	1.1 (0.2–8.2)
***CT/CTA metric***	***67 (24/36)***	***32 (147/463)***	***4.0 (2.0–8.1)***
**MRI Imaging**			
White matter disease	83 (25/30)	77 (300/388)	1.4 (0.6–3.8)
Prior imaging stroke	33 (10/30)	28 (84/304)	1.7 (0.8–3.7)
Microbleeds	23 (7/30)	16 (62/390)	1.6 (0.7–3.6)
***DWI positive***	***75 (27/36)***	***57 (262/463)***	***2.2 (1.05–4.7)***

CT/CTA metric: acute ischemia on CT, intracranial or extracranial occlusion or stenosis ≥50%. CT/CTA descriptors: white matter disease (presence of white matter hypodensities consistent with small vessel disease), prior stroke (evidence of previous cerebral infarction), aortic arch atheroma (focal area of protruding plaque ≥4 mm), ICA intraluminal thrombus (Internal carotid artery intraluminal thrombus – area of hypodensity surrounded by contrast on CT, consistent with an acute thrombus). MRI: Any white matter disease (any area of hyperintensity on the white matter on FLAIR sequence that is not part of an acute cerebral infarction), Prior stroke (evidence of prior stroke), microbleeds (small round area of hypointensity seen on gradient echo sequence).

The results for the exploratory analysis of predictors of stroke progression and recurrent stroke assessed individually rather than in the combined are shown in table 3. Only variables previously shown to be statistically significant in the original CT/CTA and MRI metrics were included to reduce the risk of a Type 1 error. The following parameters were predictive for symptom progression: ongoing symptoms at initial assessment, symptom fluctuation, intracranial occlusion, intracranial occlusion or stenosis, and the original CT/CTA metric. Imaging parameters had much less of an effect on prediction of recurrent stroke than symptom progression. Although none of these variables reached statistical significance, both intracranial occlusion/stenosis ≥50% and extracranial carotid occlusion/stenosis ≥50% showed a strong trend towards prediction of recurrence.

**Table 3:** Effect of clinical and imaging parameters on symptom progression (persistent worsening of symptoms related to the presenting event) and recurrent stroke (persistent new or worsening symptoms caused by something other than the presenting event).

Variable	Progression % (no./No.)	No Progression % (no./No.)	Hazard Ratio (95% CI)
Age 60 or greater	74 (14/19)	70 (336/480)	1.2 (0.4–3.3)
Male sex	37 (7/19)	41 (199/480)	0.8 (0.3–2.1)
Diabetes	16 (3/19)	15 (73/480)	1.04 (0.3–3.6)
Hypertension	47 (9/19)	57 (272/480)	0.69 (0.3–1.7)
Initial BP>140 systolic or 90 diastolic	68 (13/19)	74 (355/480)	0.77 (0.3–2)
Ongoing symptoms at first assessment	89 (17/19)	60 (290/480)	5.4 (1.2–23.2)
Symptom fluctuation	26 (5/19)	11 (51/480)	2.9 (1.03–8)
**Imaging Findings**			
Acute ischemia on CT	21 (4/19)	12 (57/480)	1.9 (0.6–5.8)
Intracranial occlusion or stenosis ≥50%	74 (14/19)	19 (90/480)	13.9 (5–39)
Intracranial occlusion	53 (10/19)	9 (42/480)	9.9 (4.0–24.4)
Intracranial stenosis ≥50%	26 (5/19)	6 (31/480)	4.7 (1.7–13)
Extracranial carotid artery occlusion or stenosis ≥50%	16 (3/19)	9 (44/480)	1.8 (0.5–6.3)
Original CT/CTA positive metric	79 (15/19)	33 (156/480)	7.3 (2.4–22)
DWI positive	79 (15/19)	57 (274/480)	2.7 (0.92–8)
	**Recurrence % (no./No.)**	**No Recurrence % (no./No.)**	
Age 60 or greater	88 (15/17)	70 (335/482)	3.2 (0.7–14.2)
Male sex	59 (10/17)	41 (196/482)	2.1 (0.8–5.5)
Diabetes	29 (5/17)	15 (71/482)	2.3 (0.8–6.6)
Hypertension	76 (13/17)	56 (268/482)	2.6 (0.8–7.8)
Initial BP>140 systolic or 90 diastolic	88 (15/17)	73 (353/482)	2.7 (0.6–11.7)
Ongoing symptoms at first assessment	65 (11/17)	61 (296/482)	1.1 (0.4–3.1)
Fluctuation	18 (3/17)	11 (53/482)	1.7 (0.5–5.8)
**Imaging Findings**			
Acute ischemia on CT	6 (1/17)	12 (60/482)	0.4 (0.1–3.3)
Intracranial occlusion or stenosis ≥50%	35 (6/17)	17 (80/482)	2.7 (0.99–7.2)
Intracranial occlusion	24 (4/17)	9 (48/482)	2.7 (0.9–8.3)
Intracranial stenosis ≥50%	12 (2/17)	7 (34/482)	1.7 (0.4–7.5)
Extracranial carotid artery occlusion or stenosis ≥50%	24(4/17)	9 (43/482)	3.0 (0.99–9.3)
Original CT/CTA positive metric	53 (9/17)	34 (162/482)	2.2 (0.8–5.7)
DWI positive	71 (12/17)	57 (277/482)	1.7 (0.6–4.9)

BP = Blood pressure at first assessment in the emergency department.

## Discussion

We were not able to refine or improve upon the imaging predictors for recurrent events identified in the original CATCH paper. We did however find that the presence of symptom fluctuation was predictive of recurrent events. In the multivariable model this was additive to the CT/CTA metric in the prediction of recurrent events. In our exploratory analysis of separate predictors of symptom progression and recurrence, we found that imaging was much more predictive of progression than recurrence.

Symptom progression was predicted by ongoing symptoms at initial assessment, fluctuating symptoms, and intracranial occlusion or stenosis. The relationship between symptom progression, intracranial occlusion, and fluctuation makes sense as there is the potential for ongoing cerebrovascular injury and impaired arterial blood flow in patients with intracranial occlusion. A clinical history of symptom fluctuation could be indicative of at-risk brain tissue receiving diminished perfusion near the threshold of normal functioning and may correlate with the presence of a proximal arterial occlusion or the precarious extent of active collaterals [19]. Therefore, it is reasonable to accept fluctuating symptoms has a harbinger of deterioration attributable to the same vascular territory responsible for the initial presentation but it would not necessarily reflect an increased risk of recurrent stroke in a different vascular territory. Symptom fluctuation may also identify patients with the capsular warning syndrome in the absence of a large artery intracranial occlusion [20]. This adds to work from other groups that have found that TIA patients with fluctuation are at high risk for recurrent stroke after TIA [21]–[22]. Intracranial occlusion in a population of minor stroke/TIA puts patients at high risk for symptom progression. This suggests that firstly all patients in this category should have urgent vascular imaging (including intracranial imaging) and that more aggressive treatments such as thrombolysis should be considered in these patients [23].

Although numbers of events are small and the precision of the estimates wide, it does appear that we are better at predicting symptom progression than recurrent stroke. It has previously been shown that a high proportion of patients with minor stroke had both clinical and radiological recurrences in the same vascular territory as their initial event [17], [24]. It was also found that recurrent events were rare outside of the initial vascular territory or perfusion deficit, perhaps suggesting that many cases of recurrence are in fact progression. It was surprising to find that recurrent strokes were not predicted by our imaging variables but this is perhaps reflective of the limitations of small numbers of recurrent events potentially due to aggressive early secondary prevention [5], [25]. Also many of the imaging parameters studied are likely to be more predicitive of recurrent events in the chronic phase post stroke, rather than in the first 90 days. There was however a strong trend, that both extracranial and intracranial stenosis/occlusion predicts recurrent stroke [25]–[28]. We also found that intraluminal thrombus [13] was an uncommon finding on CTA, 2.4% (12/499) and did not predict recurrent events. The lack of relationship with recurrent events is perhaps explained by the combination of low prevalence, possibly low sensitivity for its identification, and utilization of aggressive medical therapy.

There is growing evidence for patients with microbleeds being at increased risk for ischemic strokes [9]–[10]. However participants in all these studies were followed for a number of years. Our study suggests that microbleeds may not be predictive of recurrent stroke events in the short term (<3 months) following TIA or minor stroke.

A previous study found that CT evidence of acute and chronic ischemia identifies patients at high risk of stroke following TIA [7]. This study also utilized a 90-day follow up period but gathered radiographic data from chart review rather than direct examination of the original images as we did. This methodological difference may have contributed to these results supporting the predictive valve of previous ischemia on CT imaging which were not replicated in our study. Further, a recent publication indicated that the addition of CT imaging (old or new infarction or periventricular white matter disease) could increase the predictive value of the ABCD score to better identify high risk TIA patients [7]. This study arrived at different conclusions than our own regarding the value of prior ischemia and white matter disease but significant methodological differences are again present. The method of rating white matter disease and the exact timing of CT imaging related to the initial stroke event were not reported in this previous study and it is conceivable that these differences have contributed to the contrary conclusions.

The relatively small number of recurrent events identified in our patient population is a significant limitation of our study. This is particularly true when progression and recurrent stroke events are analyzed separately and the confidence intervals are wide. This is also a single centre study with a standardized CTA and this work needs to be repeated in different populations. We also used a conservative definition of clinical deterioration and the reliability of this definition has not been tested. In terms of refining the MRI parameters that predict recurrent events, we are limited by the fact that only 82% of patients had an MRI as well as a CT/CTA.

In conclusion we found that we could not improve the imaging predictors of recurrent events (combined outcome of symptom progression and recurrent stroke) by a more detailed analysis of the CT, CTA or MRI. We also found that imaging is more predictive of symptom progression rather than recurrent stroke.
